# A Case Report of Cardiobacterium hominis Endocarditis in a Pregnant Woman

**DOI:** 10.7759/cureus.9827

**Published:** 2020-08-18

**Authors:** D Sarumathi, G Anitha, Deepashree R, Rajeev Thilak C, Apurba S Sastry

**Affiliations:** 1 Microbiology, Jawaharlal Institute of Postgraduate Medical Education and Research, Puducherry, IND; 2 Cardiothoracic Surgery, Jawaharlal Institute of Postgraduate Medical Education and Research, Puducherry, IND

**Keywords:** infective endocarditis, cardiobacterium hominis, hacek group, maldi-tof

## Abstract

Infective endocarditis (IE) is an infrequent endovascular disease, which can result in significant mortality and morbidity. *Staphylococcus aureus* and viridans streptococci remain the most common etiological agent. *Cardiobacterium hominis*, a member of the HACEK (*Haemophilus* species, *Aggregatibacter* species, *Cardiobacterium hominis*, *Eikenella corrodens*, and *Kingella* species) group of fastidious gram-negative bacillus, is a part of normal upper respiratory flora and a rare cause implicated in IE. Cases of *Cardiobacterium hominis* endocarditis are being increasingly reported in last few years due to advancement in automated blood culture system such as BacT/ALERT Virtuo^®^ and identification system such as MALDI-TOF MS (matrix-assisted laser desorption/ionization time-of-flight mass spectrometry). We herein report a first case of *Cardiobacterium hominis* endocarditis in a pregnant woman at 20 weeks of gestation. Following spontaneous abortion and evacuation of the fetus, appropriate surgical intervention under heparinized condition and pathogen-directed medical intervention was initiated in this patient. This case report highlights the importance of appropriate antimicrobial therapy, which augments earlier resolution of the disease.

## Introduction

Infective endocarditis (IE) is a destructive cardiovascular infection associated with high morbidity and mortality [1,2]. The most common etiological agent implicated in IE is gram-positive bacteria such as Staphylococcus aureus [1]. The HACEK group of organisms (*Haemophilus species*, *Aggregatibacter* species, *Cardiobacterium hominis*, *Eikenella corrodens*, and *Kingella* species) is a rare cause of IE, accounting for approximately <5% of IE [3]. *Cardiobacterium hominis*, a highly fastidious gram-negative bacillus and a normal oral commensal [4], can occasionally cause IE. The identification of *C. hominis* is difficult as it grows very slowly and requires special enriched media for growth [5]. However, with the recent advancement in the diagnostic tools such as the automated blood culture system BacT/ALERT Virtuo® (bioMérieux, Marcy-l'Étoile, France) and matrix-assisted laser desorption/ionization time-of-flight mass spectrometry (MALDI-TOF MS), the cases of C. hominis endocarditis are being increasingly reported in the recent years [5]. Prompt and accurate diagnosis will help in instituting appropriate management; which includes early surgical intervention along with prolonged antimicrobial therapy [6,7]. In pregnancy, IE is extremely rare, but, when present, it contributes to significant maternal and fetal mortality [8].To the best of our knowledge, no reports of *C. hominis* IE has been reported in pregnant women. We describe a case of mitral valve IE due to *C. hominis* in a 35-year-old pregnant woman who was successfully treated with medical and surgical interventions.

## Case presentation

A 35-year-old multigravida woman at 20th week of gestation presented with new onset of chest pain and difficulty in breathing. She had left-sided retrosternal chest pain, which was insidious onset, with on and off episodes, each lasting for one to two minutes with no radiation, aggravated by heavy work and relieved with rest, along with breathlessness, palpitation, pedal edema for one month, and a history of intermittent fever for one-week duration. She did not have other comorbidities, and treatment history was not relevant.

**Figure 1:**
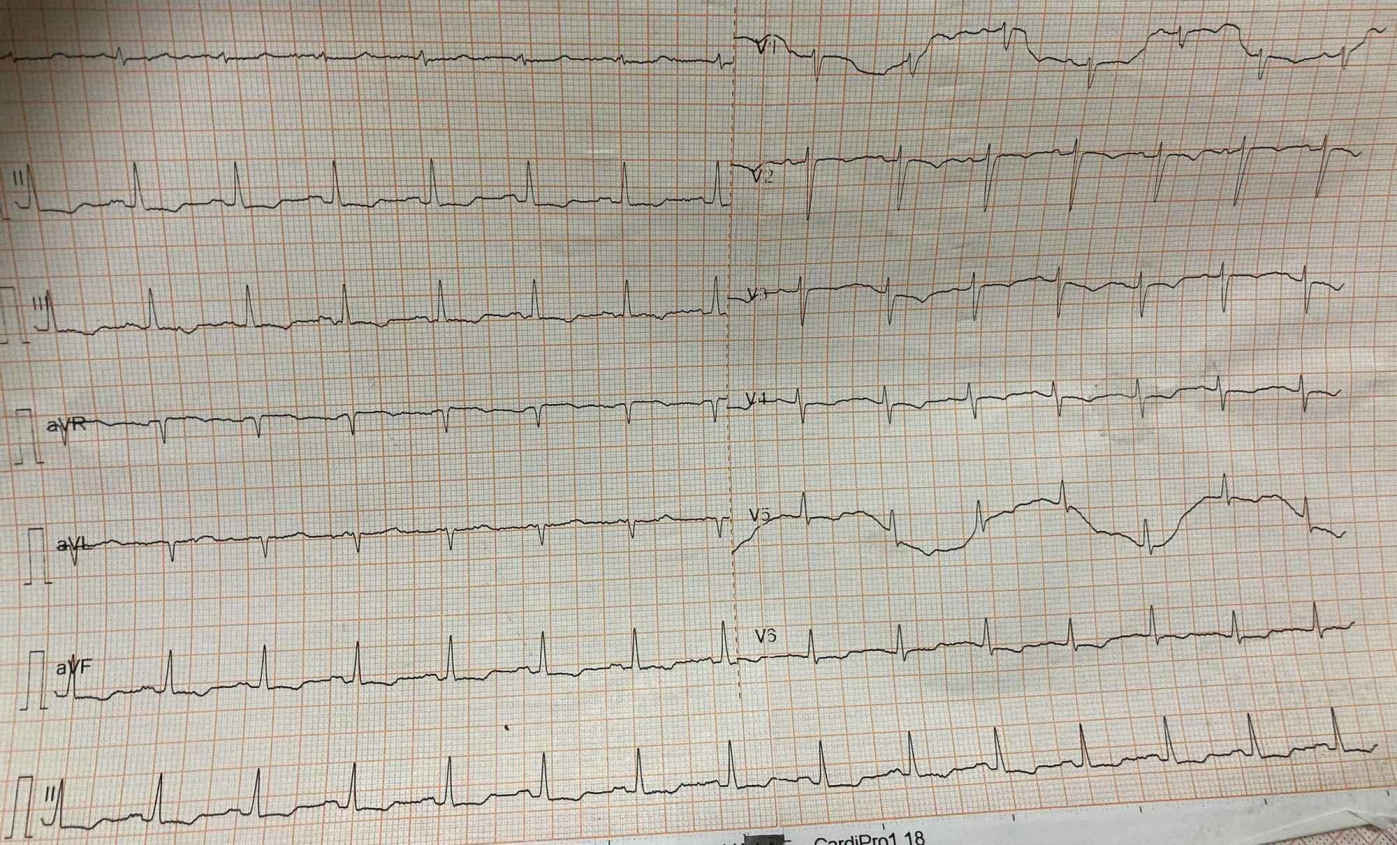
Electrocardiogram

**Figure 2:**
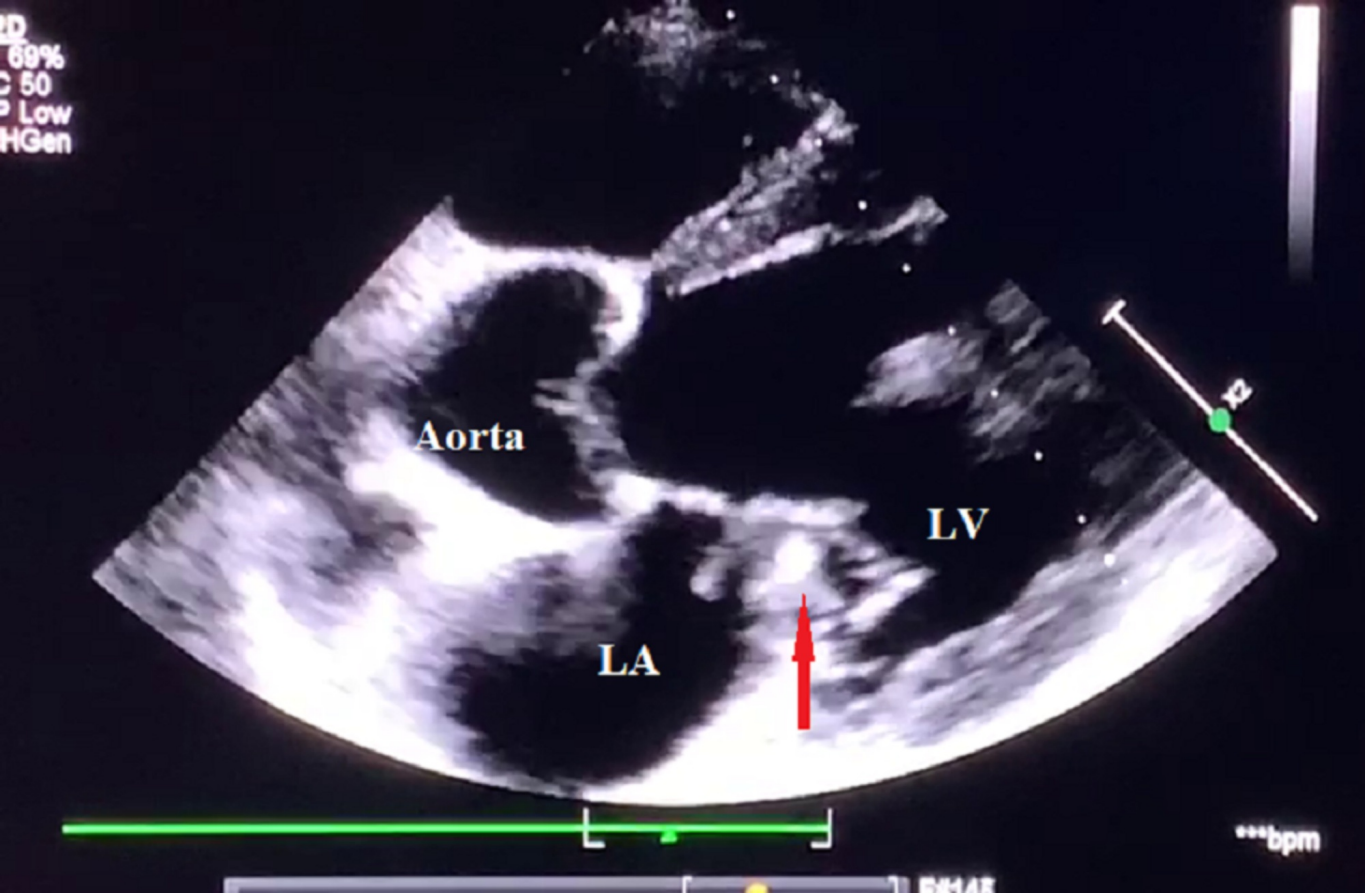
Trans-esophageal echocardiography Red arrow shows 7 x 12 mm vegetation attached to the anterior mitral leaflet.

Laboratory examination demonstrated a white blood cell count of 11,000 per microliter and normochromic normocytic anemia (Hb-5gm %). An electrocardiogram was normal (Figure 1) and a trans-esophageal echocardiography (TEE) (Figure 2) demonstrated 7 x 12 mm vegetation attached to the anterior mitral leaflet with chordal rupture, causing severe anterior mitral leaflet prolapse. There was severe eccentric mitral regurgitation (MR) and sub-valvular calcification with mitral stenosis and mild aortic regurgitation. She complained of acute pain in the left lower limb. An arterial Doppler ultrasound was performed, which showed a thrombus in the left femoral artery. Left transfemoral embolectomy was performed. As IE was suspected, the clinical team collected three pairs of blood culture in BacT/ALERT bottles before the start of antibiotics and sent for bacteriological culture. Subsequently, empirical antibiotic therapy was initiated. The bottles were immediately loaded in BacT/ALERT Virtuo automated instrument. Meanwhile, pregnancy was spontaneously aborted and fetal products were evacuated. Four out of six blood culture bottles were flagged positive after seven days of the incubation following which gram stain and subculture were performed on to blood agar and Macconkey agar. Gram stain revealed highly pleomorphic gram-negative rods of 1-3 µm in length arranged in short chains and rosettes with an irregular staining pattern. Colonies on blood agar were small, circular, flat, non-hemolytic. Biochemical tests performed from the colonies revealed catalase negative, oxidase positive, indole positive, and nitrate negative. Then the colonies were subjected to MALDI-TOF (version 3.2, bioMérieux), which subsequently was identified as *C. hominis*. The instrument had given a confidence score of 99.9% for the identification.

Based on the culture report targeted therapy comprising of ceftriaxone for four weeks combined with gentamicin for two weeks was initiated. After 14 days of pathogen-directed therapy, the blood culture was repeated and the culture turned out to be sterile. The patient clinically improved and subsequently was discharged.

## Discussion

IE, a rare but severe form of valvular heart disease, is characterized by inflammatory state of the endocardium and linings of the heart valves [1]. Multiple factors predispose patients to the development of IE, such as intravenous drug use, prosthetic heart valves, structural heart disease, prior rheumatic fever, recent dental procedure, previous endocarditis, and congenital valvular disease [9]. The diagnosis of IE is based on the modified Duke’s criteria, which incorporate information from echocardiography, history and physical examination, blood culture, and histopathology [10]. The pathogenesis involves endothelial injury triggering sterile thrombus and subsequent adhesion of bacteria, which together constitute mature vegetation. Such bacteria enter through a traumatized surface mucosa of the body, probably the oral cavity, gut, or genitourinary tract [1].

Various studies have shown *Staphylococcus aureus* as the leading organism involved in native valve endocarditis (NVE) followed by viridans streptococci, *Enterococcus*, and coagulase-negative staphylococci [1,3]. In more than 10% of IE, called as culture-negative IE, the causative agent is unknown. The classical example is the HACEK group of organisms. However, the word “culture-negative IE” is a misnomer as with the advent of sophistically automated technique it is now possible to isolate many of these fastidious organisms including HACEK. BacT/ALERT Virtuo is an advanced automated culture method that works on the same principle of BacT/ALERT. However, it offers several advantages such as automated loading and unloading of bottles without opening the doors, which helps to provide optimal incubatory condition without temperature fluctuations.

*Cardiobacterium hominis* is one among the HACEK group and is an uncommon cause of IE. It is a fastidious gram-negative coccobacillus that belongs to the family *Coriobacteriaceae* (includes three genera: *Cardiobacterium*, *Dichelobacter*, and *Suttonella*) [11]. *Cardiobacterium hominis* is considered as microaerophilic, which grows best in a humid atmosphere with an increased CO_2 _tension [12]. *Cardiobacterium hominis* is considered as a member of the normal flora of the mouth and has been recovered from the nose or throat of normal individuals [4]. It rarely causes human infections such as endovascular infections, septic arthritis, ocular infections, and neonatal sepsis. It generally follows a subacute course of IE [13]. It is of low virulence and affects previously damaged or prosthetic valves [9]. It has a strong association with aortic valve infection and is known to produce large friable vegetations [3]. Penicillin along with aminoglycosides was the standard treatment regimen given for HACEK endocarditis in the past. It is found that *C. hominis* is generally susceptible to penicillin, ampicillin, cephalosporins, fluoroquinolones, chloramphenicol, and tetracycline. Beta lactamase producing strains of *Cardiobacterium* have been described recently [9]. The American Heart Association/Infectious Disease Society of America endocarditis guidelines recommend monotherapy regimen comprising either ceftriaxone (2 g/24 hours IV/IM in one dose) or ampicillin-sulbactam for a duration of four weeks for HACEK endocarditis [14].

## Conclusions

The case report highlights the significance of a rare pathogenic agent *C. hominis* responsible for IE in a pregnant woman of 20 weeks’ gestational age. We also emphasize the importance of automated blood culture systems, especially BacT/alert Virtuo and better pathogen identification methods such as MALDI-TOF MS, which aid in the appropriate antimicrobial therapy, thereby augmenting the earlier resolution of the disease.
